# The Effect of Visible Light on Cell Envelope Subproteome during *Vibrio harveyi* Survival at 20 °C in Seawater

**DOI:** 10.3390/microorganisms9030594

**Published:** 2021-03-13

**Authors:** Maite Orruño, Claudia Parada, Vladimir R. Kaberdin, Inés Arana

**Affiliations:** 1 Department of Immunology, Microbiology and Parasitology, Faculty of Science and Technology, University of the Basque Country UPV/EHU, 48340 Leioa, Spain; maite.orruno@ehu.eus (M.O.); cbparadamorais@gmail.com (C.P.); vladimir.kaberdin@ehu.eus (V.R.K.); 2 Research Centre for Experimental Marine Biology and Biotechnology (PIE-UPV/EHU), 48620 Plentzia, Spain; 3 IKERBASQUE, Basque Foundation for Science, 48013 Bilbao, Spain

**Keywords:** *Vibrio*, seawater, starvation, visible light, membrane subproteome

## Abstract

A number of *Vibrio* spp. belong to the well-studied model organisms used to understand the strategies developed by marine bacteria to cope with adverse conditions (starvation, suboptimal temperature, solar radiation, etc.) in their natural environments. Temperature and nutrient availability are considered to be the key factors that influence *Vibrio harveyi* physiology, morphology, and persistence in aquatic systems. In contrast to the well-studied effects of temperature and starvation on *Vibrio* survival, little is known about the impact of visible light able to cause photooxidative stress. Here we employ *V. harveyi* ATCC 14126^T^ as a model organism to analyze and compare the survival patterns and changes in the protein composition of its cell envelope during the long-term permanence of this bacterium in seawater microcosm at 20 °C in the presence and absence of illumination with visible light. We found that *V. harveyi* exposure to visible light reduces cell culturability likely inducing the entry into the Viable but Non Culturable state (VBNC), whereas populations maintained in darkness remained culturable for at least 21 days. Despite these differences, the starved cells in both populations underwent morphological changes by reducing their size. Moreover, further proteomic analysis revealed a number of changes in the composition of cell envelope potentially accountable for the different adaptation pattern manifested in the absence and presence of visible light.

## 1. Introduction

*Vibrio* species are frequently used as models organisms to study the strategies developed by marine bacteria to cope with adverse and changing environments. A large number of studies have demonstrated that the survival of vibrios in the natural environment is largely determined by temperature, and some authors [1,2] have indicated that these bacteria represent an important and tangible barometer sensing the impact of climate change in marine ecosystems.

Unlike low temperatures (below 13 °C) that could often lead to vibrio’s dormancy and promote acquisition of the Viable But Non Culturable (VBNC) state in *Vibrio alginolyticus* [3], *V. cholerae* [4], *V. harveyi* [5,6], *V. parahaemolyticus* [7,8], or *V. vulnificus* [9], moderate temperatures (13 °C to 22 °C) seem to increase the capacity of vibrios to survive under starvation [10,11], thereby potentially increasing the spread of *Vibrio* species-associated diseases [12,13,14].

These effects are more profound during the summer seasons, which are also characterized by more intensive solar radiation. Several studies have reported the complex responses of aquatic bacteria exposed to photosynthetically active radiation (PAR; visible light) (400 to 700 nm). While it positively affects the physiology of autochthonous marine bacteria or bacterioplankton communities [15,16], the visible light can also decrease the culturability and viability of allochthonous species [17,18].

Photooxidative reactions attributable to the formation of reactive oxygen species (ROS) (peroxides, superoxide, hydroxyl radical, and singlet oxygen) have been considered to be the main reason of the harmful effect of light [19,20]. In marine environments, the absorption of solar radiation by the dissolved organic matter leads to the photochemical production of ROS [21], thus leading to oxidative stress frequently faced by marine vibrios.

Temperature has been demonstrated to induce changes in expression of some antioxidant enzymes [22,23] as well as those involved in DNA damage repair [24]. Kong et al. [25] found that low temperatures could lead to the loss of catalase activity while Kaberdin et al. [26] detected that genes involved in antioxidant stress responses were upregulated for *Vibrio* populations maintained at 20 °C. The above findings suggest that temperature can affect both the survival of *V. harveyi* and its resistance to oxidative stress. Despite some progress in the field, there is still very little is known about the combined effects of visible light and temperature on *Vibrio* spp. persistence in seawater [27,28,29].

The aim of the present study was to compare the survival strategy developed by *V. harveyi* during the long-term permanence in a seawater microcosm at 20 °C in the presence and absence of visible light. Given the important role of cell envelope in adaptation to changing environments [5,18,30], the study was mainly focused on analyzing the changes in cell envelope subproteome that take place during this process.

## 2. Materials and Methods

### 2.1. Vibrio harveyi Strain and Inocula Preparation

A *V. harveyi* strain ATCC 14126^T^ was used throughout this study. For inocula preparation, cells were cultured aerobically in marine broth (MB, PanReac AppliChem, Barcelona, Spain) at 26 °C with shaking (120 rpm) until they reached the stationary phase. The cells were harvested by centrifugation (4000× *g*, 4 °C, 20 min), washed three times with sterile saline solution (1.94% NaCl, *w*/*v*) and were suspended afterwards in sterile saline solution.

### 2.2. Survival Experiments

All the glass flasks used for handling *V. harveyi* cultures were cleaned with H_2_SO_4_ (96%, *v*/*v*) beforehand, rinsed with deionized water, and kept at 250 °C for 24 h to get rid of residual organic matter.

Erlenmeyer flasks containing 2 L filtered and autoclaved seawater, collected from Port of Armintza in the North of Spain (43°26′24″ N and 2°54′24″ W), were inoculated with stationary-phase *V. harveyi* cells to reach a density of 10^8^ cells mL^–1^ and incubated at 20 °C with shaking (120 rpm) in darkness and exposed to photosynthetically active radiation (PAR) up to 21 days. Illumination was provided by five Sylvania Standard F25W/30″ lamps emitting in the 400 to 700 range. The populations received a light intensity of 15.93 W m^−2^. Periodically, samples were collected in triplicate for bacterial count, determination of cell size and extraction of membrane proteins.

All the experiments were performed three times. The values presented in datasets are the means of three experiments, and the standard deviations between replicates were less than 12%. The differences between the means were calculated by a one-way analysis of variances. Probabilities that were less than (or equal to) 0.05 were considered to be significant.

### 2.3. Cell Counting and Estimation of Bacterial Size

The total number of bacteria (TNB) was determined according to the procedure described by Hobbie et al. [31]. Viable bacteria, estimated as bacteria with intact cytoplasmic membranes (MEMB+), were counted with Live/Dead BacLight^TM^ kit (Thermo Fisher Scientific Inc., Madrid, Spain) as described by Joux et al. [32]. The number of culturable bacteria (CFU) was determined by spreading cell suspensions on marine agar (MA, PanReac AppliChem, Barcelona, Spain) followed by incubation for 24 h at 26 °C and cell counting.

The length variations of *V. harveyi* cells during their survival at 20 °C were estimated via image analysis of epifluorescence preparations [33] by using an image analysis system, which included a video camera of high resolution (Hamamatsu 2400, Hamamatsu Photonics, Hamamatsu City, Japan). Digitized images of microscopic fields were analyzed by Scion Image 1.62^a^ software. In total, 200 cells were measured in each sample. The mean value (x) and the corresponding standard deviation (SD), which defined the size of the cells in the initial inoculate, were used to establish three arbitrary ranges of cell size (≤x-SD, >x-SD and ≤ x+SD, >x+SD) subsequently used to present the time-dependent changes of cell size in *V. harveyi* populations [5].

### 2.4. Isolation of Membrane Proteins by Using Sodium Carbonate Extraction

To extract membrane proteins, cells were harvested by centrifugation (8000× *g*, 40 min, 4 °C) and the pellet obtained was suspended in 10 mL of Tris-buffered saline (TBS, pH 8). The cells were collected by centrifugation again (8000× *g*, 20 min, 4 °C), the cell pellet was briefly washed with TBS, which was decanted and the cell pellet was suspended in 10 mL of TBS followed by addition of 250 μL of Protease Inhibitor Cocktail (Sigma–Aldrich, Madrid, Spain) per g of cell pellet and 90 μL of 2 mM of phenylmethylsulfonyl fluoride (PSMF, PanReac AppliChem, Barcelona, Spain), the suspensions were frozen in liquid nitrogen and stored at −80 °C.

Cells were disrupted by intermittent sonication (SONICS VibraCell^TM^ VCX130 Ultrasonic Cell Disruptor, Sonics & Materials Inc., Newtown, CT, USA) using a 6-mm-diameter probe (65% amplitude setting, 30 s on/45 s off cycles for 3 min in total). Unbroken cells and cellular debris were removed by centrifugation at 6000× *g* for 20 min at 4 °C. The supernatant fractions were stored on ice while the pellets were suspended in 10 mL of TBS and sonicated under the same conditions as specified above. This procedure was repeated at least three times.

The supernatants obtained from the same samples were combined, diluted (1:1) with 0.2 M sodium carbonate solution, incubated on ice for 1 h with gentle shaking and ultracentrifuged at 115,000× *g* for 1 h at 4 °C. The supernatants were discarded and protein pellets containing membrane proteins were suspended in 1 mL of TBS.

### 2.5. Protein Identification and Quantification

Analysis of protein samples containing membrane proteins was performed in the Proteomics Core Facility-SGIKER at the University of the Basque Country, using the protocol previously described by González-Fernández et al. [34]. Briefly, 50 μg of total protein were precipitated by using a 2-D Clean-Up kit (GE Healthcare, Bilbao, Spain) according to the manufacturer’s instructions. The pellet was suspended in 0.2% RapiGest solution (Waters Corporation, Cerdayola del Vallès, Spain), heated (85 °C, 15 min), reduced with DL-dithiothreitol (DTT, 5 mM), alkylated with iodoacetamide (15 mM) and digested with trypsin (Roche Diagnostics, Leganés, Spain; 2 μg per sample) overnight at 37 °C. RapiGest was inactivated by the addition of HCl at a final concentration of 0.5% and incubation at 37 °C for 40 min. Samples were centrifuged at 16,000× *g* for 10 min, the supernatant was collected and MassPREP Enolase Digestion Standard (Waters Corporation, Cerdayola del Vallès, Spain) was added as an internal standard for protein absolute quantification. Data independent acquisition analyses were performed in a NanoAcquity UPLC System coupled to a SYNAPT HDMS (Waters Corporation, Cerdayola del Vallès, Spain). A final amount of 0.5 μg (containing tryptic peptides and 100 fmol of MassPREP Enolase Digestion Standard) were loaded onto a Symmetry 300 C18, 180 μm × 20 mm precolumn (Waters Corporation). The precolumn was connected to a BEH130 C18 column (75 μm × 200 mm, 1.7 μm [Waters Corporation, Cerdayola del Vallès, Spain]) and peptides were eluted with a 120 min linear gradient (3 to 40%) of acetonitrile (*v*/*v*) followed by a 15 min linear gradient (40 to 60%) of acetonitrile (*v*/*v*). Mass spectra (MS) were acquired using a data independent acquisition mode (MSE) described by Silva et al. [35]. Briefly, 1 s alternate MS acquisitions were performed at low (6 eV) and high (12–35 eV ramping) collision energies and the radio frequency (RF) offset was adjusted so that the MS data were acquired from m/z 350 to 1990. [Glu1]-fibrinopeptide B (Sigma–Aldrich) at a concentration of 100 fmol/μL sprayed through the NanoLockSpray source and sampled every 30 s. The obtained spectra were processed with ProteinLynx Global Server v2.4 Build RC7 (Waters Corporation) using the doubly protonated monoisotopic ion of [Glu1]-fibrinopeptide B for mass correction. Protein identification was carried out by using the embedded database search algorithm of the program [36] and a *Vibrio harveyi* UniProt Knowledgebase (UniProtKB) (version 2020_06, 4950 sequences). For protein identification, the following parameters were adopted: carbamidomethylation of C as a fixed modification; N-terminal acetylation, N and Q deamidation, and M oxidation, as variable modifications; 1 missed cleavage and default automatic precursor and fragment error tolerance. A maximum false positive rate of 5% was allowed. Absolute protein quantification based on peak area intensity of peptide precursors was automatically calculated by ProteinLynx Global Server using Enolase peptides as an internal standard [37]. A total of 648 proteins were confirmed by finding at least three protein-derived peptides in the tryptic digest, 327 proteins were detected in at least two biological replicates and were subsequently used for absolute quantification. Individual absolute quantification values were normalized versus the total protein amount present in the sample. Proteins with a significant (*p* < 0.05, t-test) increase (>1.5 fold) or decrease (<0.5 fold) in their relative abundance with respect to the initial time (P0) were considered to be differentially affected by survival conditions.

UniProt (http://www.uniprot.org/) and KEGG: Kyoto Encyclopedia of Genes and Genomes (http://www.genome.jp/kegg/) databases were used to verify the name and possible function of the proteins (accessed on 1 November 2020). The subcellular localization of many polypeptides annotated as membrane-associated proteins with known functions was further scrutinized by searching for the cognate membrane-binding domains with the PSORTb 3.0 program [38].

## 3. Results

### 3.1. Analysis of V. harveyi Persistence at 20 °C

**Figure 1:**
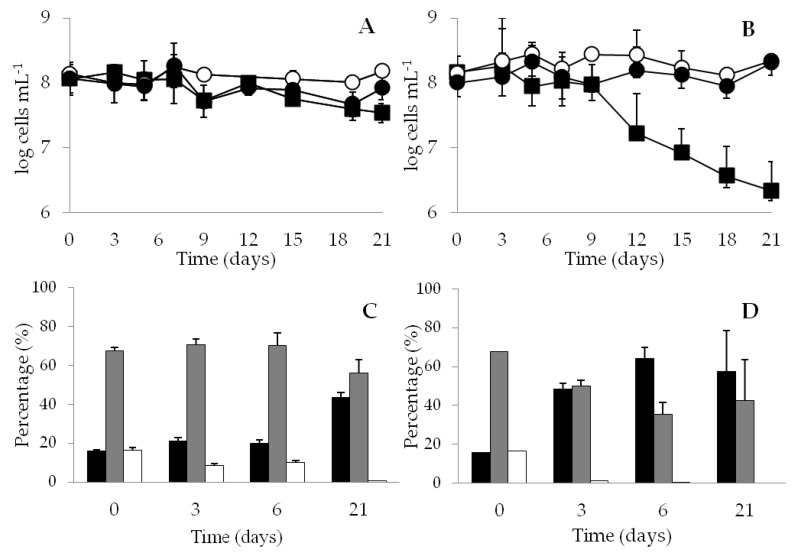
*V. harveyi* ATCC 14126^T^ survival in seawater (starvation) at 20 °C in dark (**A**,**C**) or in the presence of illumination (**B**,**D**). A and B, variations of total (○), viable (●) and culturable bacteria in Marine Agar (■) obtained during 21 days. C and D, variations in cell size distribution (█ ≤ 0.91 μm; █ > 0.91- ≤ 1.74 μm; 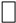 > 1.74 μm). The data are mean values from three independent experiments with errors bars representing the standard deviations calculated.

The variations in integrity, viability, culturability, and cell size distribution of *V. harveyi* populations maintained at 20 °C under nutrient scarcity (i.e., incubation in seawater microcosms) are shown in Figure 1. The numbers of total (TNB) and viable (MEMB+) bacteria remained practically unchanged throughout the experimentation time regardless of PAR irradiation. However, the number of culturable cells (CFU) declined approximately 0.53 and 1.83 log after 21 d of incubation in the absence and presence of illumination, respectively (Figure 1A,B). The significant loss of culturability for population exposed to PAR, along with the preservation of cell viability, indicated that the major part of the population (98.51%) had likely acquired the VBNC phenotype at the end of the incubation time.

The size of the starved *V. harveyi* cells varied along the survival process, in fact, the cells reduced considerably their length during incubation, from a medium length of 1.93 µm at the beginning of the experiments to 0.97 or 0.92 µm after 21 d of incubation in the absence or presence of illumination, respectively. These phenotypical changes led to the appearance of cells with the coccoid-like morphology associated with the VBNC state in *Vibrio* species [39]. The length reduction was more profound when the experiments were carried out under illumination. The fraction of shorter cells (length ≤ 0.91 µm) increased nearly 3.6 times during exposure to visible light and about 2.7 times in darkness when compared to the initial values (Figure 1C,D), ultimately reaching 43.5 and 57.5%, respectively. Moreover, the cells with a length exceeding 1.74 µm were not found after 21 days of incubation under both conditions.

### 3.2. Changes of Membrane Subproteome during Permanence at 20 °C

From survival assays carried out in darkness or upon exposure to visible light, the samples were collected at different incubation times: immediately after inoculation (P0), 6 days (P1), and 21 days (P2). Proteins detected in at least two biological replicates, whose biological functions were previously defined or could be inferred by homology, were selected for further analysis. The dataset of proteins contained a high number of predicted cytosolic proteins (35.5%). Some of them belonged to cytosolic subunits of membrane protein complexes or were annotated as proteins that can transiently be associated with the membrane [5]. The above properties may explain the presence of these “cytosolic” proteins in the membrane fraction. After determining the composition of the membrane subproteomes, the identified proteins were sorted according to their biological functions and grouped to form the following categories of proteins involved in: (i) maintenance of cell structure, (ii) transport, (iii) bioenergetics, (iv) signal transduction, (v) protein synthesis, degradation and turnover, or other (vi) miscellaneous functions.

The proteins that did not show any significant variation in abundance (i.e., they were not upregulated [>1.5-fold] or downregulated [<0.5-fold]) during the survival experiments are listed in Table 1.

This group includes proteins involved in (i) maintaining the structure of cell envelope (e.g., lipoproteins [D0XEL2_VIBH1, D0XD95_VIBH1], components of the β-barrel assembly machinery [BAM] complex, membrane protein insertase YidC and rod shape-determining protein MreB); (ii) transmembrane transport (ion transporters as OmpU or D0XAK6_VIBH1 porin, vitamin B12 transporter BtuB, maltose operon periplasmic protein [MalM] and others) and in protein translocation (YajC, SecA, and SecD subunits) and secretion (Type II secretion system core protein G, a TolC family protein or multidrug resistance protein MdtA); as well as (iii) proteins whose function is related to bioenergetics (namely, different subunits of ATP synthase, cytochrome b or subunits of Na(+)-translocating NADH-quinone reductase); (v) protein biogenesis (HflC and HflK proteins); and (vi) translation (elongation factors EF-Tu and EF-G).

In addition to proteins listed in Table 1, there was a group of proteins whose level was affected by experimental conditions (Table 2). In other words, we found that the level of numerous proteins was altered after 21 d of starvation at 20 °C both in the absence and presence of PAR irradiation.

Namely, some components of phosphotransferase systems (PTS) (D0X8N6_VIBH1, D0XD90_VIBH1), TatA protein translocase, cytochrome c5, so-called methyl-accepting chemotaxis proteins (D0XEK4_VIBH1, D0XEY1_VIBH1, D0X9J1_VIBH1, D0XEC5_VIBH1, D0XHW4_VIBH1, D0XGG1_VIBH1, D0X5R4_VIBH1, D0X9F5_VIBH1, and D0XCQ6_VIBH1) and flagellin became undetectable (Table 2), whereas the level of YhcB readily declined after 21 d. On the contrary, only a few proteins (e.g., mechanosensitive ion channel MscS, bacterioferritin and catalase-peroxidase, see Table 2) undetectable in the initial inoculates (time P0) were detected after long-term starvation at 20 °C under both conditions. These proteins were detected earlier (6 d) in populations maintained under illumination.

In addition, there was a group of membrane proteins differentially affected by incubation in the presence vs. absence of illumination. For instance, several proteins related to bioenergetic (e.g., cytochrome c oxidase subunit CcoO, cytochrome c4, ubiquinol-cytochrome c reductase iron sulfur subunit, ubiquinol-cytochrome c reductase cytochrome c1) and others (penicillin-binding protein activator LpoA and proteases, D0XAK5_VIBH1 and ATP-dependent Zn protease) were downregulated only in populations that were exposed to PAR.

*Vice versa*, several proteins, in particular those related to transport of phosphate and glucose, one isoform of ATP synthase subunit beta, OmpK, OmpA-like protein D0X6J9_VIBH1, and ATP-dependent zinc metalloprotease FtsH, were downregulated (or undetectable) only in the populations maintained in darkness. The upregulation of several proteins was likewise light-dependent. Namely, while some proteins (ATP synthase subunit c, subunit I of cytochrome d ubiquinol oxidase or NAD (P) transhydrogenase subunit beta) were upregulated in darkness, the same polypeptides were undetectable in the populations exposed to PAR. Similarly, illumination of starved *V. harveyi* cells for 21 days led to an increase in the level of some structural and transport-related proteins including the outer membrane protein Slp, YcfL peptidoglycan-associated lipoprotein D0XFJ5_VIBH1, general secretion pathway protein D, outer membrane protein TolC and agglutination protein D0XI94_VIBH1.

## 4. Discussion

The life style and persistence of microorganisms in natural aquatic systems are greatly dependent on diverse abiotic and biotic stress factors (e.g., suboptimal salinity and pH, temperature up- and downshifts, nutrient availability, solar radiation, predation, etc.). A number of studies, which were aimed at addressing the effect of these environmental factors, previously employed *V. harveyi* as a model organism. Although the individual impact of some stress factors on *V. harveyi* is well characterized [40,41], little is known about their joined action.

Here we studied the combined effects of nutrient limitation, temperature, and visible light on *V. harveyi* adaptation in seawater microcosms. A typical and well-documented survival response of several *Vibrio* species under nutrient limitation is the acquisition of the VBNC state [4,8,9,42,43], which is more frequently observed at low temperatures rather than at temperatures ranging from 13 to 22 °C [9,26,44,45]. Consistently, we found that, unlike in the experiments carried out at 4 °C [5], *V. harveyi* strain ATCC 14126^T^ populations did not acquire the VBNC state after at least three weeks of incubation in seawater (nutrient scarcity, darkness) at 20 °C. Thus, our data indicate that, similar to the key role of temperature in adaptation of *V. parahaemolyticus* [46] or *V. vulnificus* [10,47], it also determines *V. harveyi* survival responses. Moreover, the persistence of *V. harveyi* populations was accompanied by a progressive reduction in cell size. This observation supports the idea that the initial response of *V. harveyi* to starvation leads to morphological changes rather than an immediate transition to the VBNC state [5,26].

In addition to nutrient availability and temperature, exposure to visible light is another important stress factor known for its contribution to *Vibrio* growth and survival [27,28,29]. To compare the long-term adaptation of *V. harveyi* in the absence and presence of illumination, experiments were also carried out upon exposure of *V. harveyi* populations to visible light. Our data demonstrate that illumination with visible light not only accelerates the bacterial size reduction but also decreases cell culturability, thus suggesting the cell entry into the VBNC state. However, the effect of visible light on transition to the VBNC state might be less profound than that in other bacteria, in which the acquisition of this phenotype can occur within a few days (e.g., in *Escherichia coli* [48,49] or *Enterococcus faecalis* [48,50]) or even hours (e.g., in *Pseudomonas aeruginosa* [51]).

As exposure to visible light can provoke oxidative stress, the prolong persistence of *Vibrio* spp. population could be due to their ability to activate protective mechanisms mitigating the damaging effects of light. In fact, Rees et al. [52] speculated on the protective role of bacterial luminescence against oxidative stress. Consistently, other authors have indicated that bacterial bioluminescence may play an important role in detoxification of reactive oxygen species [53,54] and in stimulating DNA repair [55,56]. Another important response to oxidative stress involves catalase overproduction. It has been described for different *Vibrio* species subjected to abiotic stress [57,58,59]. In addition, recent studies described overexpression of other enzymes conferring protection against the toxic effects of H_2_O_2_ and reactive oxygen species during *V. harveyi* permanence in seawater at different temperatures [24,26]. Therefore, our observation that catalase peroxidase becomes detectable in populations exposed to visible light earlier (i.e., at time P1) than in those lacking illumination could indicate a role of this enzyme in sustaining *V. harveyi* resistance to visible light.

*V. harveyi* survival is determined by the capacity of this bacterium to respond to changing environment by reprogramming gene expression, thus affecting the entire proteome. Owing to the essential role of cell envelope in bacterial adaptation to stress, the second part of this study was focused on determining the stress-related changes in *V. harveyi* membrane subproteome and discussing their possible contribution to cell resistance to stress. Analysis of cell envelope subproteome revealed that the level of some membrane proteins with key roles in maintenance of cellular structure, transport, and bioenergetic processes remained unchanged until 21 d (Table 1). Moreover, some of those proteins (i.e., OmpW, maltose operon periplasmic protein, vitamin B12 transporter BtuB, several ATP synthase subunits, Na(+)-translocating NADH quinone reductase subunits or Hflk protein) were also maintained in VBNC populations induced during exposure to cold temperature [5]. Therefore, these proteins appear to constitute a pool of proteins inherently present in the viable (culturable or nonculturable) cells under starvation. Additionally, the level of many structural proteins detected in this study was preserved or even increased under stress. They include lipoproteins, Bam factors and other outer membrane proteins (e.g., OmpW porin [60]) apparently essential for maintaining the integrity of the outer cell membrane throughout the survival process.

MreB (Table 1) is another protein whose level remained nearly the same during the survival process. This protein plays an important role in cell shape maintenance and division [61]. While sequence analysis predicts that it is a cytoplasmic protein, there is some evidence that suggests the transient association of this protein with the membrane [62]. Moreover, Chiu et al. [63] demonstrated that the MreB protein could be detected close to the membrane in starved cells. Although previous work had shown that MreB became undetectable in the membrane fraction during the first 12 h of permanence at 4 °C [5], we did not see any significant time-dependent changes in MreB levels for populations incubated at 20 °C in the presence or absence of illumination (present study). These results suggest that MreB association with the membrane (and therefore its cellular localization) in the starved cells are likely dependent on temperature.

The permanent presence of other membrane proteins (Table 1) is likely linked to their essential functions (e.g., protein transport) during starvation. Indeed, our data revealed the constant presence of proteins (D0X124_VIBH1, D0X520_VIBH1) that were components of Types I and II secretion systems. Moreover, the concentration of some components (D0X523_VIBH1, D0X7A6_VIBH1, D0XI94_VIBH1) has even increased in the illuminated populations after 21 days. Concerning the Sec-mediated transport in *V. harveyi,* the abundance of SecA, SecD, and YajC proteins also remained unaltered along survival, while TatA became already undetectable after 6 days of starvation, thereby suggesting that nutrient limitation favored Tat-independent secretion. Unlike the mechanisms affecting protein secretion in *V. harveyi*, *Brucella suis* response to starvation appears to limit the Sec-dependent transport possibly to reduce the overall metabolic activity and energy consumption [64]. Similarly, *Campylobacter jejuni* persistence in tap water at different temperatures [65] has been reported to support the Tat-dependent (rather than Sec-dependent) transport.

Elongation factors are essential bacterial proteins, and EF-TU has been described as a cytoplasmic chaperone [42,66] involved in protein synthesis and other cellular processes [67,68]. The higher abundance of EF-TU in the subproteomes of the stressed cells [5,18] and its upregulation upon exposure to stress [5,69] could imply the involvement of EF-TU in cell adaptation. Nevertheless, the lack of significant variations in the level of these proteins in populations examined in the present study does not support the general role of these protein factors in bacterial adaptation to stress.

Besides the continuous presence of many proteins apparently essential for both the normal growth and cell survival (Table 1), there was a group of polypeptides differentially affected by starvation (Table 2). Variations in their levels could be attributable to stress adaptation induced by nutrient deprivation, and subsequent energy and carbon depletion. In particular, we found that multiple methyl-accepting chemotaxis proteins became rapidly undetectable in *V. harveyi* populations maintained in seawater at 20 °C regardless of exposure to visible light, thus mimicking *V. harveyi* response to starvation at 4 °C [5]. Similarly, the level of flagellin declined after 21 days. These results agree with previous observations obtained with starved *Vibrio* S14 cells by Malmcrona-Friberg et al. [70]. They found that most cells lost motility under starvation and suggested that the chemosensory system could be shutdown already after first 24 h of starvation. Likewise, our findings are also consistent with the results of Stretton et al. [71], who showed the detachment of flagellum during the first days of starvation and argued that due to high energy cost of synthesis, assembly, and function of flagellum, the transition to the non-motile (but metabolically active) state would be more beneficial for cells under starvation. In a similar study, Chen and Chen [72] likewise demonstrated that *V. vulnificus* motility diminished along the time of permanence under nutrient scarcity conditions. Moreover, some authors [73,74,75] revealed that starvation not only leads to the loss of motility but also increases cell adhesion. Therefore, the loss of chemotactic activity and motility observed in our study for *V. harveyi* populations under starvation could be an important strategy enabling to save energy and ensure cell survival under stress.

Dissolved iron concentrations in open ocean surface waters typically stay below 0.2 nM [76], thus establishing iron-limiting conditions for marine organisms. Several authors [77,78] indicated that the control of iron homeostasis and responses to oxidative stress are interdependent. In other words, iron is not only an essential element for bacterial growth but it is also a toxic metal able to promote the formation of reactive oxygen species (ROS). They cause oxidative stress, consequently elevating the level of catalase peroxidase. In previous work, the success of *V. harveyi* permanence in seawater microcosms at 4 °C was linked to iron homeostasis involving bacterioferritin during the entry into the VBNC [5]. Regarding the populations exposed to starvation at 20 °C (present study), bacterioferritin, which was undetectable at the beginning of experiments, becoming expressed even though no culturability loss was detected.

A similar expression pattern was observed for the mechanosensitive ion channel protein MscS. It seems likely that in addition to its role in coping with osmotic stress, this protein is also involved in cell wall repairing to protect against sustained stress [79] stimulated by visible light.

Taken together, our results demonstrate that *V. harveyi* adaptation to starvation at 20 °C induces morphological changes leading to cell size reduction and acquisition of the coccoid-like morphology, apparently triggering the acquisition of the VBNC state by the cell exposed to visible light. This finding suggests that the exposure to visible light along with variations in temperature, salinity, and others, might promote the *V. harveyi* entry to the VBNC state in aquatic systems. Moreover, several studies have proposed that the VBNC cells can potentially preserve their capacity to elicit infections [6,39,80]. Further analysis of cell envelope subproteome revealed that a number of membrane proteins playing the key roles in maintenance of major cell envelope functions constitute a pool of proteins continuously present in viable (culturable and nonculturable) *V. harveyi* ATCC14126^T^ populations exposed to stress. The presence of these proteins enables to sustain the key functions of the membranes, such as selective permeability and transport. In contrast, nutrient depletion leads to the loss of proteins involved in cell mobility and chemotaxis. In addition, starvation could potentially affect iron homeostasis largely dependent in the stressed populations on bacterioferritin. Likewise, as exposure to visible light potentially increases oxidative stress, there was a continuous presence of catalase and peroxidase proteins in the long starved cells. Taken together, our proteomic data indicate that adjustments in the cell envelope subproteome were more profound in the case of populations exposed to visible light.

## Figures and Tables

**Table 1:** Membrane proteins of *V. harveyi* ATCC 14126^T^ whose level did not show a significant change (>1.5 or <0.5 fold change) after 6 days (P1), and 21 days (P2) of starvation in seawater at 20 °C with respect to initial values (P0).

Protein Accession Number	Location ^a^	Category ^b^	Protein Name (Uniprot)
D0XEL2_VIBH1	OM	S	Major outer membrane lipoprotein
D0XD95_VIBH1	CM	S	Lipoprotein
D0X8I2_VIBH1	OM	S	Outer membrane protein assembly factor BamA
D0X8E2_VIBH1	OM	S	Outer membrane protein assembly factor BamC
D0XDX1_VIBH1	OM	S	Outer membrane protein assembly factor BamD
D0XEA2_VIBH1	CM	S	Membrane protein insertase YidC
D0XAE4_VIBH1	Cyt	S	Rod shape-determining protein MreB
D0X6I2_VIBH1	OM	T	OmpU
D0X5G7_VIBH1	OM	T	OmpW
D0XAK6_VIBH1	OM	T	Gram-negative porin family protein
D0XAX8_VIBH1	OM	T	Vitamin B12 transporter BtuB
D0XI69_VIBH1	P	T	Maltose operon periplasmic protein (MalM)
D0XDJ8_VIBH1	CM	T	Preprotein translocase subunit YajC
D0X7E4_VIBH1	CM	T	Protein translocase subunit SecA
D0XDJ7_VIBH1	CM	T	Protein translocase subunit SecD
D0X520_VIBH1	CM	T	Type II secretion system core protein G
D0X596_VIBH1	IM	T	Multidrug resistance protein MdtA
D0XI24_VIBH1	OM	T	Type I secretion outer membrane, TolC family protein
D0X6W0_VIBH1	CM	T	Na+/proline symporter
D0XAD2_VIBH1	OM	T	MSHA biogenesis protein MshL
D0X9U0_VIBH1	CM	T	LemA family protein
D0XE90_VIBH1	Cyt	B	ATP synthase subunit alpha
D0X9V8_VIBH1	Cyt	B	ATP synthase subunit alpha
D0XE88_VIBH1	Cyt	B	ATP synthase subunit beta
D0XE92_VIBH1	Cyt	B	ATP synthase subunit b
D0X7C0_VIBH1	CM	B	Cytochrome b
D0X872_VIBH1	CM	B	Na(+)- translocating NADH-quinone reductase subunit A
D0X873_VIBH1	CM	B	Na(+)-translocating NADH-quinone reductase subunit B
D0X877_VIBH1	CM	B	Na(+)-translocating NADH-quinone reductase subunit F
D0X602_VIBH1	CM	B	NAD(P) transhydrogenase subunit alpha
D0X8Q1_VIBH1	CM	B	Succinate dehydrogenase iron-sulfur subunit
D0XAW2_VIBH1	CM	SDT	HflC
D0XAW1_VIBH1	CM	SDT	HflK
D0X8K6_VIBH1	CM	SDT	Modulator of FtsH protease HflK
D0XAQ5_VIBH1	Cyt	O	Elongation factor Tu (EF-Tu)
D0XCD5_VIBH1	Cyt	O	Elongation factor G (EF-G)

^a^ OM, outer membrane; CM, cytoplasmic membrane; Cyt, cytosolic; P, periplasmic according to PSORTb 3.0 program. ^b^ Category: S, structural; T, transport; B, bioenergetics; ST, signal transduction; SDT, Synthesis, degradation and turnover of proteins; O, others.

**Table 2:** Changes in the level of membrane proteins of *V. harveyi* ATCC 14126^T^ subjected to starvation in seawater at 20 °C. The data are presented for the initial population (time P0) and populations analyzed after starvation of *V. harveyi* for 6 (P1) and 21 (P2) days in the absence (−) and presence (+) of illumination.

Protein Accession Number	Loc ^a^	Cat ^b^	Protein Name	P0 ^c^	Illumination			
					(−)		(+)	
					P1	P2	P1	P2
D0X8N6_VIBH1	CM	T	PTS system N-acetylglucosamine-specific IIABC component	D	ND	ND	ND	ND
D0XD90_VIBH1	CM	T	PTS system trehalose-specific IIBC component	D	ND	ND	ND	ND
D0X553_VIBH1	CM	T	Sec-independent protein translocase protein TatA	D	ND	ND	ND	ND
D0X593_VIBH1	?	B	Cytochrome c5	D	ND	ND	ND	ND
D0XEK4_VIBH1 D0XEY1_VIBH1	CM	O	Methyl-accepting chemotaxis proteins	D	ND	ND	ND	ND
D0X9J1_VIBH1 D0XEC5_VIBH1 D0XHW4_VIBH1 D0XGG1_VIBH1 D0X5R4_VIBH1 D0X9F5_VIBH1 D0XCQ6_VIBH1	CM	O	Methyl-accepting chemotaxis proteins	D	ND	ND	D	ND
D0XD78_VIBH1	CM	S	Tail-specific protease	D	ND	ND	D	ND
D0XB91_VIBH1	Ex	O	Flagellin	D	D	ND	ND	ND
D0X7B5_VIBH1	CM	S	Protein YhcB	D	D	0.49	D	0.22
D0X6W2_VIBH1	CM/Cyt	T	Bifunctional protein PutA	D	0.17	0.18	0.26	0.11
D0XFE0_VIBH1	CM	B	Cytochrome c oxidase subunit CcoO	D	D	D	D	ND
D0X7B9_VIBH1	CM	B	Ubiquinol-cytochrome c reductase iron sulfur subunit	D	D	D	D	ND
D0X7C7_VIBH1	P	S	Penicillin-binding protein activator LpoA	D	D	D	D	ND
D0XAK5_VIBH1	?	SDT	Protease	D	D	D	D	ND
D0X545_VIBH1	P	B	Cytochrome c4	D	D	D	ND	ND
D0X955_VIBH1	CM	T	Multidrug resistance protein MexA	D	D	D	ND	ND
D0XFH9_VIBH1	?	SDT	ATP-dependent Zn protease	D	D	D	ND	ND
D0XE93_VIBH1	CM	B	ATP synthase subunit c	D	D	5.50	ND	ND
D0XFI8_VIBH1	CM	B	Cytochrome d ubiquinol oxidase, subunit I	D	D	4.29	ND	ND
D0X601_VIBH1	CM	B	NAD(P) transhydrogenase subunit beta	D	D	2.65	D	ND
D0X7C1_VIBH1	CM	B	Ubiquinol-cytochrome c reductase cytochrome c1	D	D	D	D	0.11
D0XFD9_VIBH1	CM	B	cbb3-type cytochrome c oxidase subunit	D	D	D	D	0.06
D0XEK0_VIBH1	CM	T	Phosphate import ATP binding protein PstB	D	D	ND	D	D
D0XEK3_VIBH1	P	T	Phosphate ABC transporter periplasmic phosphate-binding protein	D	ND	ND	D	3.36
D0X9E5_VIBH1	Cyt	SDT	ATP dependent Clp protease ATP binding subunit ClpA	D	ND	ND	D	D
D0X9N4_VIBH1	Cyt	SDT	ATP dependent Clp protease ATP binding subunit ClpX	D	ND	ND	D	D
D0XCF5_VIBH1	CM	T	PTS system glucose-specific IIBC component	D	D	0.38	D	D
D0X6J9_VIBH1	OM	T	OmpA–like protein	D	D	0.40	D	D
D0X9V6_VIBH1	CM	B	ATP synthase subunit beta	D	0.07	0.18	D	D
D0XCA0_VIBH1	CM	SDT	ATP-dependent zinc metalloprotease FtsH	D	D	0.48	D	D
D0X861_VIBH1	OM	O	Outer membrane protein OmpK	D	D	0.32	D	D
D0X8R9_VIBH1	OM	S	Outer membrane protein slp	D	D	D	D	2.06
D0X9I8_VIBH1	OM	S	Putative YcfL protein	D	D	D	D	2.92
D0XFJ5_VIBH1	OM	S	Peptidoglycan-associated lipoprotein	D	D	D	D	2.43
D0X523_VIBH1	OM	T	General secretion pathway protein D	D	D	D	D	2.20
D0X7A6_VIBH1	OM	T	Outer membrane protein TolC	D	D	D	D	2.75
D0XI94_VIBH1	OM	T	Agglutination protein	D	D	D	D	4.12
D0XCR3_VIBH1	CM	T	Mechanosensitive ion channel protein MscS	ND	ND	D	D	D
D0XAQ6_VIBH1	Cyt	O	Bacterioferritin	ND	ND	D	D	D
D0X8W1_VIBH1	Cyt	O	Catalase peroxidase	ND	ND	D	D	D

^a^ Loc, Location: OM, outer membrane; CM, cytoplasmic membrane; Cyt, cytosolic; P, periplasmic; Ex, extracellular; ?, unknow, according to PSORTb 3.0 program. ^b^ Cat, Category: S, structural; T, transport; B, bioenergetics; ST, signal transduction; SDT, Synthesis, degradation and turnover of proteins; O, others. ^c^ D, present or no significant changes with respect to the previous time; ND, Not detected; and numbers corresponding to fold changes of the analyzed proteins with respect to those in the initial populations. The fold changes that are more than 1.5 and less than 0.5 indicate significant increases and decreases in protein level, respectively.

## Data Availability

The data presented in this study are available on request from the corresponding author.
